# Long-term benefit of Microbiota Transfer Therapy on autism symptoms and gut microbiota

**DOI:** 10.1038/s41598-019-42183-0

**Published:** 2019-04-09

**Authors:** Dae-Wook Kang, James B. Adams, Devon M. Coleman, Elena L. Pollard, Juan Maldonado, Sharon McDonough-Means, J. Gregory Caporaso, Rosa Krajmalnik-Brown

**Affiliations:** 1 Biodesign Swette Center for Environmental Biotechnology, Arizona State University, Tempe, AZ 85287 USA; 2 Biodesign Center for Fundamental and Applied Microbiomics, Arizona State University, Tempe, AZ 85287 USA; 3 School for Engineering of Matter, Transport and Energy, Arizona State University, Tempe, AZ 85287 USA; 4 Integrative Developmental Pediatrics, Tucson, AZ 85701 USA; 5 Center for Applied Microbiome Science, Pathogen and Microbiome Institute, Northern Arizona University, Flagstaff, AZ 86001 USA; 6 Department of Biological Sciences, Northern Arizona University, Flagstaff, AZ 86001 USA; 7 School of Sustainable Engineering and the Built Environment, Arizona State University, Tempe, AZ 85287 USA; 8 Present Address: Department of Civil and Environmental Engineering, The University of Toledo, Toledo, OH 43606 USA

## Abstract

Many studies have reported abnormal gut microbiota in individuals with Autism Spectrum Disorders (ASD), suggesting a link between gut microbiome and autism-like behaviors. Modifying the gut microbiome is a potential route to improve gastrointestinal (GI) and behavioral symptoms in children with ASD, and fecal microbiota transplant could transform the dysbiotic gut microbiome toward a healthy one by delivering a large number of commensal microbes from a healthy donor. We previously performed an open-label trial of Microbiota Transfer Therapy (MTT) that combined antibiotics, a bowel cleanse, a stomach-acid suppressant, and fecal microbiota transplant, and observed significant improvements in GI symptoms, autism-related symptoms, and gut microbiota. Here, we report on a follow-up with the same 18 participants two years after treatment was completed. Notably, most improvements in GI symptoms were maintained, and autism-related symptoms improved even more after the end of treatment. Important changes in gut microbiota at the end of treatment remained at follow-up, including significant increases in bacterial diversity and relative abundances of *Bifidobacteria* and *Prevotella*. Our observations demonstrate the long-term safety and efficacy of MTT as a potential therapy to treat children with ASD who have GI problems, and warrant a double-blind, placebo-controlled trial in the future.

## Introduction

The human gut and brain interact in complex ways, and abnormal conditions in the gut may predispose individuals to neurodevelopmental disorders^1,2^. Individuals with Autism Spectrum Disorders (ASD)^3^, Parkinson’s disease^4^, and Alzheimer’s disease^5^, for example, have been known to experience chronic gastrointestinal (GI) symptoms as a common co-occurring medical condition, suggesting the presence of a gut-brain axis. Hallmayer *et al*.^6^ investigated 192 twin pairs and found that both genetic and environmental factors contribute to the etiology of ASD. The gut microbiome represents an important environmental factor that may exert an influence on symptoms, and a growing number of research groups have observed that children with ASD have distinctive gut microbiomes compared to neurotypical children^7–11^. Moreover, multiple mouse studies have reported that gut microbes and their metabolites can impact behavior through the gut-brain axis, including for ASD^12–14^.

Effective treatments for ASD include behavioral therapy, speech and social therapy, and dietary/nutritional/medical treatments, but no medical treatment has been approved to treat core symptoms of ASD^15^, such as social communication difficulties and repetitive behaviors. Considering the link between the gut and brain, modulating the gut microbiome by antibiotics, probiotics, prebiotics, and/or fecal microbiota transplant (FMT) could be a viable therapeutic option. In FMT, a large diversity and number of commensal microbes from a healthy donor are used to transform a dysbiotic gut microbiome into a healthy microbiome. In fact, FMT is the most effective therapy to treat recurrent *Clostridium difficile* infection^16^ and has shown varying levels of success for treating other GI disorders^17^, which has drawn attention to the method for use beyond GI-associated disorders^18^. Previously, we performed a pioneering open-label modified-FMT trial with an intensive combination called Microbial Transfer Therapy (MTT) consisting of two-week vancomycin treatment followed by a bowel cleanse and then high dose FMT for 1–2 days and 7–8 weeks of daily maintenance doses along with a stomach-acid suppressant, administered to children with ASD and chronic gastrointestinal problems^19^. After this 10-week MTT treatment and an eight-week follow-up observation period (18 weeks in total), we observed an 80% reduction in GI symptoms and a slow but steady improvement in core ASD symptoms. At the same time, we learned that gut microbial diversity, including potentially beneficial microbes, significantly increased after MTT^19^. Two years after this original clinical trial was completed, we re-evaluated the participants to determine whether observed improvements in behavior and GI symptoms persisted, and to ascertain the long-term impact of MTT on the gut microbiome of the study participants.

## Results and Discussion

### Improvements in GI and ASD symptoms remained two years after the MTT stopped

**Figure 1:**
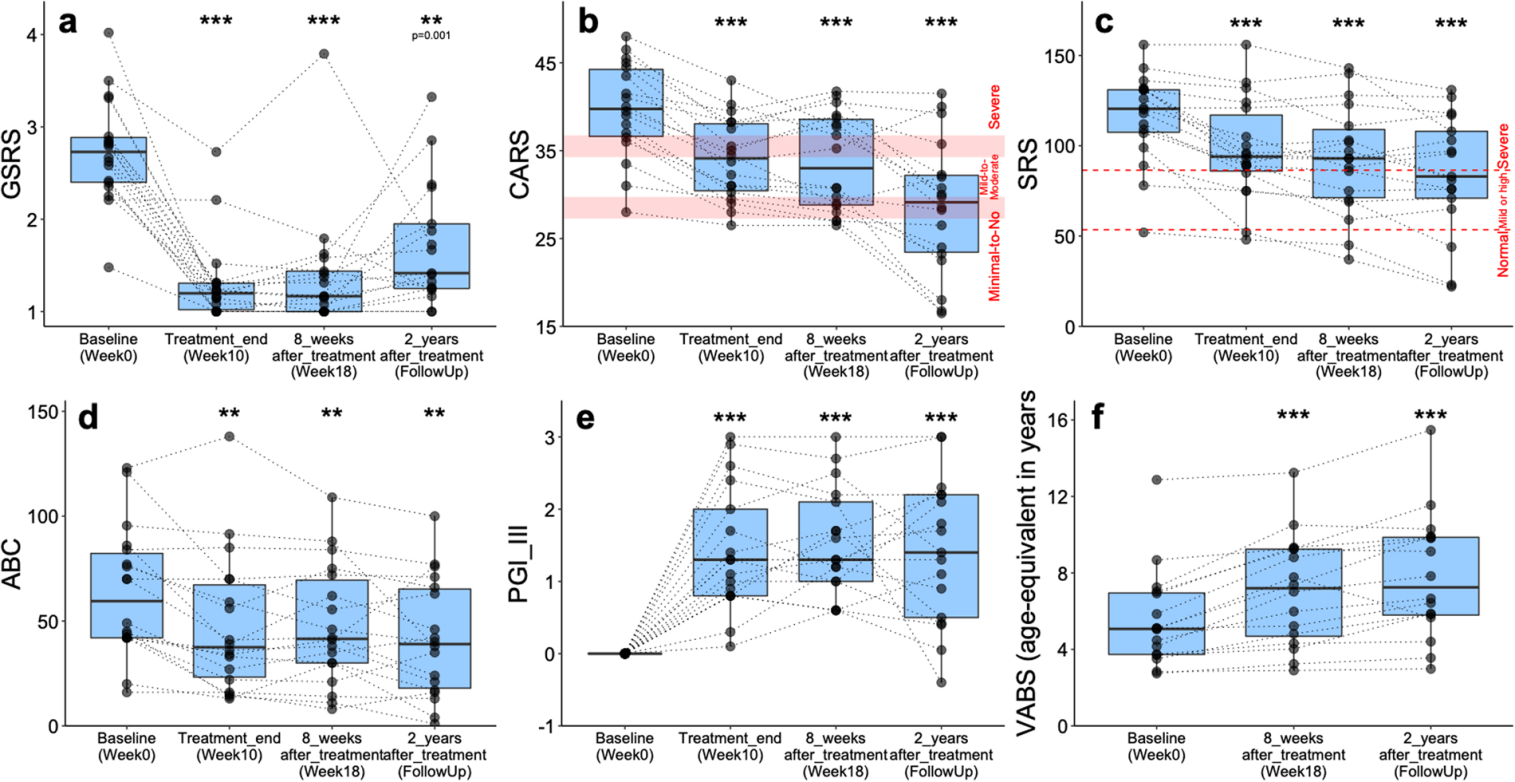
Changes in GI- and ASD-related symptoms of 18 children with ASD at two-year follow-up after treatment stopped. Asterisks (at the top of the box plot) indicate whether individuals (at each time point) have significantly changed since pre-treatment (Week 0 of original Phase 1 trial). Based on two-tailed Wilcoxon signed-rank test*, ns* indicates not significant, single asterisk indicates *p* < 0.05, double asterisks indicate *p* < 0.01, triple asterisks indicate *p* < 0.001. See also Supplementary Figs S1–S3.

**Figure 2:**
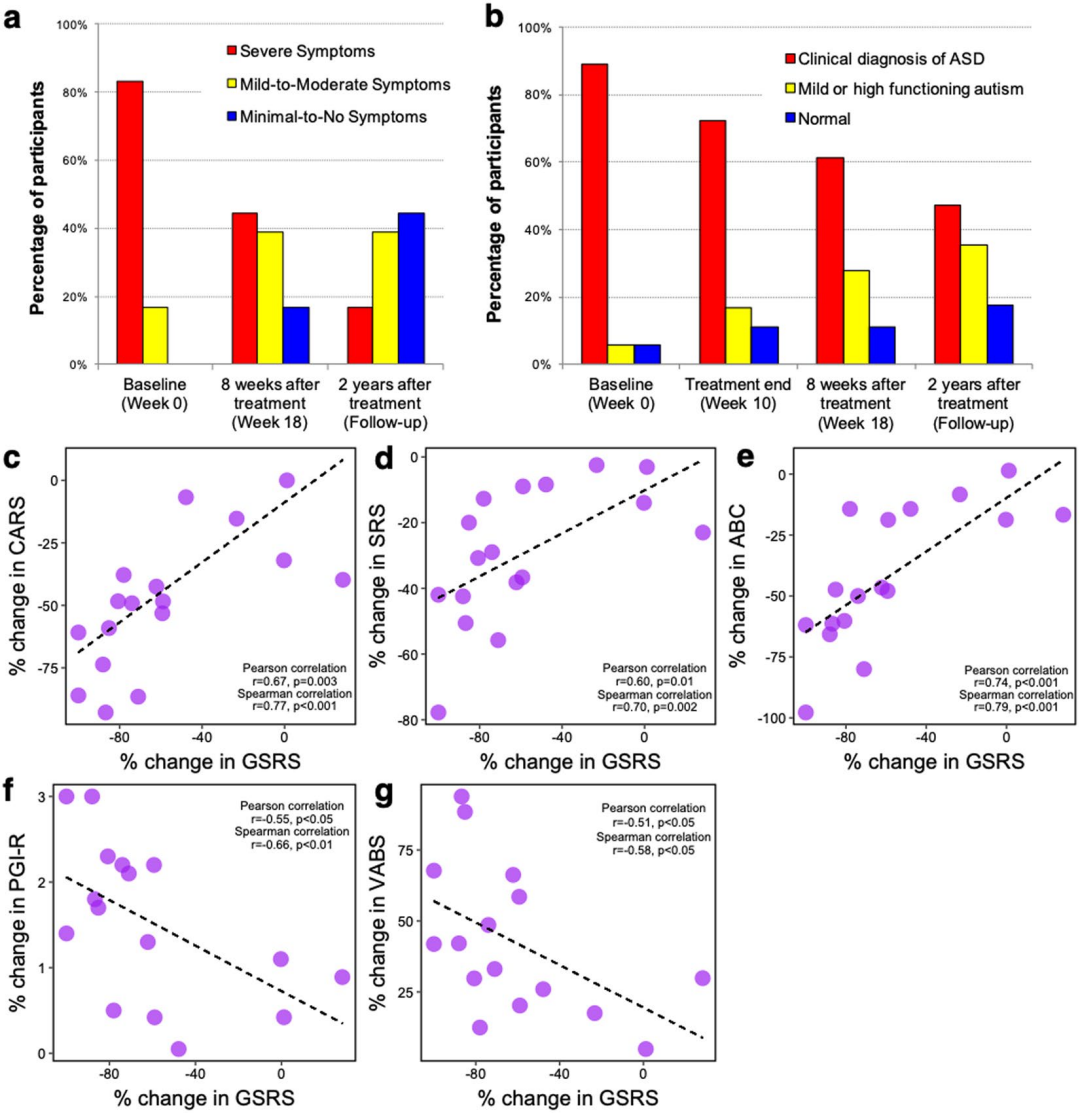
CARS and SRS diagnostic category for ASD at baseline, 8 weeks after treatment, and two-year follow-up after treatment stopped. (**a**) For CARS, Minimal-to-No Symptoms (15–29.5 for ages less than 13; 15–27.5 for ages 13 or older), Mild-to-Moderate Symptoms (30–36.5 for ages less than 13; 28–34.5 for ages 13 or order), and Sever Symptoms (37 and higher for ages less than 13; 35 and higher for ages 13 or order)^54^. (**b**) For SRS, Normal (0–53), Mild or High Functioning autism (54–86), Clinical diagnosis of autistic disorder, Asperger’s disorder, or more severe cases of Pervasive developmental disorder not otherwise specified (PDD-NOS) (>87)^55^. (**c**–**g**) Strong and significant correlations between improvements in GI symptoms (GSRS) and behavior symptoms based on % changes in 2 years.

Two years after the MTT was completed, we invited the 18 original subjects in our treatment group to participate in a follow-up study, and all provided informed consent. We performed the same GI and behavior tests that we employed previously^19^. 12 of 18 participants made some changes to their medication, diet, or nutritional supplements, but these changes were well documented and were mostly minor (Supplementary Table S1). We note that due to the open-label nature of this initial trial, all of the assessments are subject to placebo effect, however the long-term improvements we observed here are promising. Two years after treatment, most participants reported GI symptoms remaining improved compared to baseline (Fig. 1a and Supplementary Fig. S1). The improvement was on average 58% reduction in Gastrointestinal Symptom Rating Scale (GSRS) and 26% reduction in % days of abnormal stools (Daily Stool Record or DSR) relative to baseline, and this result is similar to what we observed at the end of treatment. The improvement in GI symptoms was observed for all sub-categories of GSRS (abdominal pain, indigestion, diarrhea, and constipation, Supplementary Fig. S2a) as well as for all sub-categories of DSR (no stool, hard stool, and soft/liquid stool, Supplementary Fig. S2b), although the degree of improvement on indigestion symptom (a sub-category of GSRS) was reduced after 2 years compared with weeks 10 and 18. This achievement is notable, because all 18 participants reported that they had had chronic GI problems (chronic constipation and/or diarrhea) since infancy, without any period of normal GI health (Supplementary Table S2). The families generally reported that ASD-related symptoms had slowly, steadily improved since week 18 of the Phase 1 trial, and this was consistent with the data reported in Fig. 1b–f. Based on the Childhood Autism Rating Scale (CARS) rated by a professional evaluator, the severity of ASD at the two-year follow-up was 47% lower than baseline (Fig. 1b), compared to 23% lower at the end of week 10. At the beginning of the open-label trial, 83% of participants rated in the severe ASD diagnosis per the CARS (Fig. 2a). At the two-year follow-up, only 17% were rated as severe, 39% were in the mild to moderate range, and 44% of participants were below the ASD diagnostic cut-off scores (Fig. 2a). The parent-rated Social Responsiveness Scale (SRS) assessment revealed that 89% of participants were in the severe range at the beginning of the trial, but the percentile dropped to 47% at the two-year follow-up (Fig. 2b), with 35% in the mild/moderate range and 18% below the cut-off for ASD. For the parent-rated Aberrant Behavior Checklist (ABC), total scores continued to improve, and were 35% lower relative to baseline (versus 24% lower at the end of treatment, relative to baseline; Fig. 1d). The Parent Global Impressions-III (PGI-III) scores remained similar to the scores at the end of treatment (week 10) of the open-label (Fig. 1e). The Vineland Adaptive Behavior Scale (VABS) equivalent age continued to improve (Fig. 1f), although not as quickly as during the treatment, resulting in an increase of 2.5 years over 2 years, which is much faster than typical for the ASD population, whose developmental age was only 49% of their physical age at the start of this study. Moreover, we observed improvement in behaviors in most sub-categories (Supplementary Figs S2c,d, and S3 for ABC, SRS, and VABS, respectively).

Overall, the most substantial improvements observed were on the CARS assessments, which was conducted by a professional evaluator and is less susceptible to placebo-effect^20^. CARS is a stable and consistent diagnostic tool with high predictive validity^21^ and has been used to evaluate participants before and after therapeutic interventions in multiple studies^20,22,23^. For the follow up CARS, the evaluator collected current information based on each question’s unique criteria. After the interview was complete for each question, the evaluator reviewed the information initially collected at baseline and used it for calibrating the final evaluation.

### Improvements in GI and ASD symptoms were significantly correlated

We performed statistical analyses to assess whether improvements in GI and ASD severity were correlated. As shown in Fig. 2c–e, percentage changes in CARS, SRS, and ABC scores were positively correlated with percent changes in GSRS scores (Spearman correlation test, 2-tailed *p* < 0.005 and r > 0.7), implying that GI relief provided by MTT may ameliorate behavioral severity in children with ASD, or vice versa, or that both may be similarly impacted by another factor. Another GI assessment, DSR, however, showed that there was no significant correlation. Although the direction of the influence is not clear, a potential clinical link between GI and behavior severity is consistent with what previous studies have reported^24,25^.

### ASD fecal bacterial diversity was higher two years after the MTT stopped

**Figure 3:**
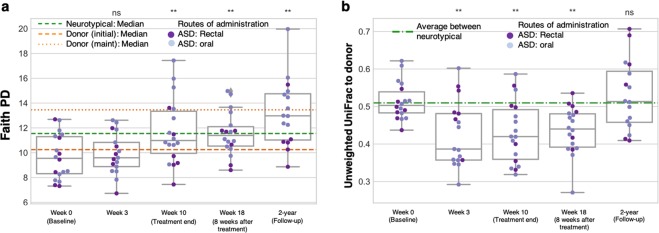
Stool microbiota assessments at two-year follow-up after treatment stopped. (**a**) Faith’s phylogenetic diversity (PD) in the microbiota of 18 children with ASD as measured from stool samples. *Orange lines* indicate median PD of the donor samples (*dashed line* represents initial donor samples (*n* = 5), and *dotted line* represents maintenance dose samples (*n* = 2)), and *green line* indicates median PD of 20 neurotypical controls at week 0. *ns* indicates not significant, *single asterisk* indicates *p* < 0.05, *double asterisks* indicate *p* < 0.01, *triple asterisks* indicate *p* < 0.001 (two-tailed Wilcoxon signed-rank test comparing weeks 3, 10, and 18 and two-year to week 0 values). (**b**) Unweighted UniFrac distances between ASD gut microbiota and most relevant donor sample (initial donor sample at weeks 0 and 3, most recent maintenance dose sample at weeks 10 and 18, and 2 years). *Green line* indicates the median interpersonal variation between neurotypical controls and illustrates that prior to treatment the difference in gut microbiota composition between MTT recipients and donors was on the order of normal interpersonal variation. Statistics are the same as those used in (**a**). See also Supplementary Figs S5 and S6.

16 out of 18 original ASD participants provided an additional fecal sample two years after the open-label trial. Based on 16S ribosomal RNA (rRNA) gene amplicon sequencing analysis, most participants maintained higher gut microbiota diversity two years after treatment relative to baseline. Interestingly, for many individuals, the bacterial diversity was higher at two years than at the week 18 follow up as measured by Faith’s Phylogenetic Diversity (Fig. 3a and Supplementary Fig. S4a) and Observed OTUs (Supplementary Fig. S5a). Considering low gut bacterial diversity in individuals with ASD^26^ and other human disorders^27–29^, an increase in diversity after MTT may reflect that MTT intervention successfully transformed gut environment into a healthier status and led to a long-term benefit on GI and behavior symptoms.

Upon completion of the original MTT treatment, we observed that the unweighted UniFrac distance^30^ between the gut microbiota of MTT recipients and their corresponding donors was smaller than before treatment, suggesting some engraftment of the donor microbiome into the recipients by MTT^19^. Interestingly, two years after the trial, the recipients were as different from the donor microbiome as they were pre-treatment as measured by unweighted UniFrac distance (Fig. 3b, Supplementary Fig. S4b) and several other metrics of community dissimilarity (Supplementary Fig. S5b–e). This suggests that the recipients didn’t retain completely the donated microbiome, but rather retained some features of it such as increased overall diversity, and increase in some important microbes such as *Prevotella*, while finding a new state.

### Bifidobacterium and Prevotella relative abundances remained higher in feces of participants with ASD two-years after MTT stopped

**Figure 4:**
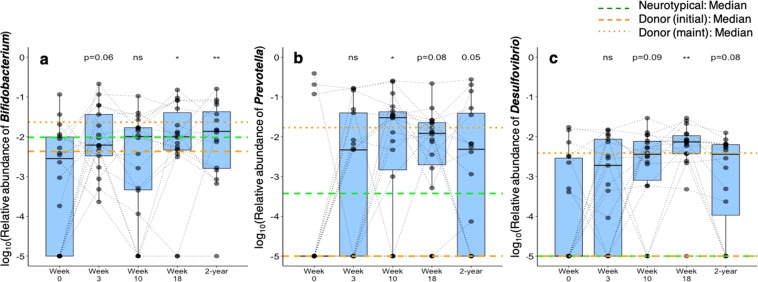
Changes in relative abundances of *Bifidobacterium*, *Prevotella*, and *Desulfovibrio*. *ns* indicates not significant, *single asterisk* indicates *p* < 0.05 and *double asterisks* indicate *p* < 0.01 (two-tailed Wilcoxon signed-rank test comparing weeks 3, 10, and 8 and two-year to week 0 values). *Orange lines* indicate median of the donor samples (*dashed line* represents initial donor samples, and *dotted line* represents maintenance dose samples), and *green line* indicates median of 20 neurotypical controls at week 0.

Three taxa that were noticeably enhanced in MTT recipients at the conclusion of the original clinical trial^19^ were revisited during the two-year follow-up. Notably, compared to baseline, median relative abundances of *Bifidobacteria* and *Prevotella* increased 4-fold and 712-fold at week 10, and 5-fold and 84-fold at two years, respectively (Fig. 4a,b). *Desulfobivrio* relative abundance decreased since week 18 (Fig. 4c), but at the two-year follow-up was still marginally higher compared to baseline (two-tailed Wilcoxon signed-rank test, *p* = 0.07) and higher than neurotypical controls (two-tailed Mann-Whitney U test, *p* < 0.05). An increase in *Prevotella* after MTT is noteworthy, since its lower abundance in feces of children with ASD compared with neurotypical children has been confirmed in two different cohorts^26,31^. A recent study also found reduced levels of *Prevotella* in the oral microbiome of children with ASD^32^. *Prevotella* may be involved in butyrate production^33^, a key nutrient for the intestinal epithelial cells^34^. In addition, its co-occurrence with *Desulfovibrio* may reflect a synergistic advantage to outcompete other commensal microbes that utilize mucin as nutrients^35^, although more research is needed on how their ecological niche in mucin desulfation could contribute to an integrity of gut epithelial cells^36^ as well as to the improvement on GI and behavior symptoms we observed. Further mechanistic studies with multi-omic approaches are warranted to define the roles of *Prevotella* and *Desulfovibrio* in the context of autism.

### Further research with a placebo-control arm is warranted

To the best of our knowledge, long-term follow-up studies are rare for medical treatment of individuals with ASD. In treatments with vancomycin^37^ or phytochemical sulforaphane^38^, benefits were lost within two or four weeks, respectively, of the treatments being discontinued. Thus, the long-term benefits observed here two years after MTT stopped are very encouraging, and MTT-driven gut microbiota transformation seems robust and long-lasting for the treatment of ASD. Despite steady and continuous improvement in behaviors over two years, we must underscore that the original clinical trial and current follow-up study are open-label trials without a control for placebo effect. Autism symptoms are relatively stable over time without a major intervention: for example, a trajectory study with 345 children with ASD showed that more than 80% of participants with ASD retained unexpectedly stable core symptoms severity over 8 to 12 years^39^. The VABS observations indicate that the improvements in adaptive behaviors observed here were substantially more than expected for children with ASD over two years. A limitation of this study is that 12 out of 18 participants made one or more changes to their medications, nutritional supplements, and diets between the end of the original MTT trial and the two-year follow-up since the treatment stopped (Supplementary Table S1). As described in detail in the methods section, participants were asked to rate the perceived effectiveness on GI and ASD symptoms (on a scale of 0–4) caused by changes in medications, diet, or nutritional supplements. Although the scale on the perceived effectiveness is still subjective and difficult to interpret, low scores received (1.1 for GI and 0.8 for ASD symptoms) suggest that these treatments on average could have only “slight effect”. Thus, it appears that most of the changes observed were probably due to the MTT, although we still need follow-up studies to understand whether the improvement by MTT were solely from vancomycin, MoviPrep, Prilosec, Standardized Human Gut Microbiota (SHGM), or a combination of these individual factors. For example, some participants in our study could have GI symptoms that were acid-peptic in nature, and their improvements on GI symptoms might be solely attributed to the administration of stomach-acid suppressant (Prilosec)^40^. We hypothesize that MTT may also be beneficial for children with ASD who do not have obvious GI symptoms but have low diversity of gut bacteria, as our previous study^26^ found that most children with ASD had low gut bacterial diversity, regardless of whether they have GI problems.

Here, we also would like to address a potential study limitation interpreting the improvement on GI symptoms after MTT, since these heterogeneous GI symptoms could reflect a wide range of underlying etiological GI pathologies. Although we reviewed medical histories to exclude children with known gastrointestinal diagnoses (such as ulcerative colitis, Crohn’s Disease, celiac disease, eosinophilic gastritis, or similar conditions)^19^, we did not conduct additional GI diagnostic evaluations, which is a limitation of this study. Thus, we want to underscore need of follow-up studies embracing more thorough examination of participants’ GI pathologies in order to better understand effectiveness of MTT.

## Conclusions

In summary, all 18 participants with ASD were re-evaluated two years after MTT treatment stopped, and we observed significant improvements both in GI and behavior symptoms as compared with baseline measurements collected at the beginning of the original open-label trial. GI benefits were mostly maintained from the end of treatment, and autism symptoms were reported to have improved significantly since the end of treatment. Changes in gut microbiota persisted at two years, including in overall community diversity and relative abundances of *Bifidobacteria* and *Prevotella*. These encouraging observations demonstrate that the intensive MTT intervention is a promising therapy for treating children with ASD who have GI problems. We recommend future research including double-blind, placebo-controlled randomized trials with a larger cohort.

## Methods

### Ethics approval and consent to participate

The protocol for the original treatment study was approved by FDA (Investigational new drug number 15886) and the Institutional Review Board of Arizona State University (ASU IRB Protocol #: 00001053), as described in Kang *et al*.^19^. All research methods were performed in accordance with the relevant guidelines and regulations. For the follow-up study described in this paper, a new IRB protocol was approved by the IRB of Arizona State University (ASU IRB Protocol # 00004890). The follow-up study was only open to the 18 children with ASD who participated in the original Phase 1 trial study, and no further inclusion or exclusion criteria was applied to what we employed in the original trial^19^. For the consent process, we contacted past participants and their parents and explained the study in detail by phone and/or email, and provided them with a copy of the parent permission form and the child assent form. After the informed consent process, all 18 families agreed to participate and signed written parent permission and assent forms. We maintained confidentiality of participants results by de-identifying all samples for the entire analyses. The participant’s name and identifiers were removed and not used in all sections of the manuscript, including supplementary information. The trial was registered at the ClinicalTrials.gov (NCT02504554) on March 30, 2015.

### Study objective

The purpose of the study was to conduct a 2-year follow-up on children with ASD who participated in the original study and to determine long-term safety and efficacy of the Microbial Transfer Therapy (MTT). In brief, the previous study was designed to perform an open-label clinical trial for children with ASD and moderate to severe GI problems (ages 7–17 years)^19^. Eighteen children were enrolled in the previous study and treated with a combination of vancomycin (to reduce pathogenic bacteria) for 2 weeks, MoviPrep (a bowel cleanse to remove vancomycin and remaining bacteria), Prilosec (a proton pump inhibitor in order to reduce stomach acidity and to increase the survival rate of high and maintenance doses of SHGM), and Standardized Human Gut Microbiota (gut bacteria from healthy donors) for 7–8 weeks^41^. Participants were monitored during the 10 weeks of treatment, and again 8 weeks after MTT stopped. There were no major adverse effects, and all 18 participants completed the study. Most of the children had substantial reduction of their GI and ASD symptoms, and those improvements remained at the follow-up at 8 weeks after the treatment ended. Further information on the previous study design and outcomes are described in Kang *et al*.^19^. In this current study, we followed up past participants approximately 2 years since the treatment stopped, and requested parents to collect one stool sample from their child in order to investigate changes in bacterial composition. After the study ended, 6 participants did not make any changes in their treatments for the next two years, and 12 participants made one or more changes (Supplementary Table S1). The changes (and the number of participants making those changes) included dietary changes (gluten-free/casein-free diet (1), ketogenic diet (1), and removing eggs(1)), GI treatments (magnesium citrate (2), digestive enzymes (1), dulcolax (1), enteragam (1), probiotics (2)), medication changes (Abilify (1), birth control (1), IVIG (1), Lamictal (1), lithium (1), Tenex (1), thryroid (1), and Vyvanse (1), and nutritional supplements (amino acids (1), multivitamins (2), Bravo yogurt (1), citruline (1), fish oil (1), GABA (1), l-theanine (1), magnesium (1), Oraimmune (1), and Rerum (1). Participants were asked to rate the perceived effectiveness of each treatment on a scale of 0–4 for both GI and ASD symptoms, where 0 = “No effect”, 1 = “Slight benefit”, 2 = “Moderate benefit”, 3 = “Good benefit”, and 4 = “Great benefit”.

### Assessments of gastrointestinal and autism-related symptoms

To be consistent with the previous assessments^19^, we evaluated GI symptoms using a revised version of Gastrointestinal Symptom Rating Scale (GSRS) that employs 7-point Likert scale^42^ in 5 domains: Abdominal Pain, Reflux, Indigestion, Diarrhea, and Constipation. We also collected Daily Stool Records (DSR) that rates the stool using the Bristol Stool Form scale (1 = very hard, 7 = liquid) over 14 days. For the assessments of autism and related symptoms, we also employed the same assessments that we used in the previous study including the Parent Global Impressions–III (PGI-III)^43^, the Childhood Autism Rating Scale (CARS), the Aberrant Behavior Checklist (ABC), the Social Responsiveness Scale (SRS), and the Vineland Adaptive Behavior Scale II (VABS-II). The CARS evaluation was conducted by the same professional evaluator who conducted the previous CARS evaluation, and parents assessed the GSRS, DSR, PGI-III, ABC, SRS, and VABS-II at approximately 2 years after the therapy stopped.

### Microbial DNA extraction and next generation sequencing

In order to be consistent with the previous work in Kang *et al*.^19^, we employed the same DNA extraction assay, library preparation, and sequencing methods. We extracted microbial genomic DNA from stool samples using a PowerSoil^®^ DNA Isolation Kit (Mobio Carlsbard, CA) and constructed 16S rRNA library for MiSeq Illumina platform according to the protocol from Earth Microbiome Project (http://www.earthmicrobiome.org/protocols-and-standards/16s/). The barcoded primer set 515f -806r was used for pair-ended sequencing to target the 16 s rRNA V4 region^44^. Library preparation and sequencing work were performed at the Microbiome Analysis Laboratory in the Biodesign Institute of Arizona State University (http://krajmalnik.environmentalbiotechnology.org/microbiome-lab.html).

### Microbiome bioinformatics

The 16S rRNA gene data from our previous sequencing run was generated from four Illumina MiSeq runs, and the follow-up data (new to this study) was sequenced on a single MiSeq run. To ensure consistent processing across all of these runs, and to enable the use of the most recent bioinformatics methods for microbiome analysis, we processed all five of these sequencing runs together in a single QIIME 2 analysis^45^.

Analyses were performed on the forward reads only, to retain as many sequences as possible (i.e., to not discard sequences where forward and reverse reads could not be joined). Sequence quality control was performed with two different processing pipelines, both implemented in QIIME 2.

The first processing pipeline used DADA2^46^ via the q2-dada2 QIIME 2 plugin (we’ll refer to this as the DADA2 pipeline). In the DADA2 pipeline, the first 10 bases were trimmed from reads and reads were truncated at 150 bases prior to processing with the dada2-single method, as recommended by the DADA2 authors. All other DADA2 parameters were left with their default settings. DADA2 was run on a per-sequencing run basis using the same parameters for all runs of DADA2. The resulting feature tables were merged, such that we had one master “feature table” containing counts of features on a per sample basis, where our features were unique 16S rRNA gene sequence variants. The “feature sequence” that defined each feature was the unique 16S rRNA gene sequence variant. Microbiome analysis results based on this processing pipeline are presented in Figs 3, 4 and Supplementary Figs S4, S5.

The second processing pipeline used the quality filtering approach described in Bokulich *et al*.^47^ as implemented in the q2-quality-filter plugin using a min-quality of 20 (all other parameters were defaults). We will refer to this pipeline as the Q20 pipeline. Sequence reads were subsequently clustered into 97% OTUs using open-reference OTU picking as implemented in the q2-vsearch plugin (which uses vsearch^48^ for clustering). Again, we had one master “feature table” containing counts of features on a per sample basis, where in this case our features were 97% OTUs. The “feature sequence” that defined each feature was the OTU centroid sequence. As an additional quality control step, features that were only observed in a single sample and features that could not be assigned to a phylum against the Silva database^49^ (described further below) were filtered from the feature table. Microbiome analysis results based on this processing pipeline are presented in Supplementary Fig. S6.

Following both the DADA2 and Q20 pipelines, feature sequences were aligned with mafft^50^ (wrapped in the q2-alignment plugin), high entropy positions were filtered from the resulting alignment^51^, an unrooted tree was constructed with FastTree^52^ (wrapped in the q2-phylogeny plugin), and the tree was rooted using midpoint rooting. Taxonomy was assigned to each feature sequence against the Silva 119 database using a Naïve Bayes classifier implemented in the q2-feature-classifier plugin^53^. Feature tables were rarefied to 5,298 sequences per sample, and diversity metrics were computed using the q2-diversity plugin.

For complete details on the bioinformatics methods, including the versions of all software and dependencies, and all commands and parameter settings, readers can view the provenance data contained in the Supplementary File S1. This information can be viewed visually at https://view.qiime2.org.

The specific values of the microbiome richness and distance metrics differ between the DADA2 and Q20 bioinformatics pipelines. For example, Faith’s Phylogenetic Diversity is considerably lower with the DADA2 pipeline, likely because it does a much better job at raw sequence quality control (as described in the original DADA2 paper). Despite this, it is encouraging to observe that the different quality control and feature definition pipelines illustrate the same trends in microbiome composition and richness.

### Statistical analysis

We assumed the data as non-normally distributed because of a relatively small sample size, and performed nonparametric analyses of Mann-Whitney U-test, Wilcoxon signed-rank test, and Spearman correlation test. We reported all *p* values from two-tailed tests, and accepted *p* values lower than 0.05 as significant. For the boxes in Figs 1, 3 and 4, the cross line in the box indicates median values and the bottom and top edges of the box indicate the 25^th^ and 75^th^ percentiles of the sample.

## Supplementary information

Supplementary Figures S1-S6

Supplementary Table S1

Supplementary Table S2

Supplementary File S1

## Data Availability

The 16S rRNA gene sequence reads are available in the open-source sequence data repository ‘Qiita’ with the study ID number 10532 (https://qiita.ucsd.edu) and the NCBI SRA under BioProject ID PRJNA529598. For details on obtaining and installing QIIME 2, see https://qiime2.org. QIIME 2 is open source and free for all use. The analyses presented in Fig. 3 and Supplementary Figs S4–S6 were performed using Jupyter Notebooks. These notebooks are available at https://github.com/caporaso-lab/autism-fmt1.
