# Supplementary material for: Long-term benefit of Microbiota Transfer Therapy on autism symptoms and gut microbiota
Source: Sci Rep. 2019 Apr 9;9:5821. doi: 10.1038/s41598-019-42183-0 (PMC6456593; doi:10.1038/s41598-019-42183-0)
Supplement: Supplementary file 1 — Supplementary Figures S1-S6 [file 41598_2019_42183_MOESM1_ESM.pdf]

Supplementary Information

Long-term benefit of Microbiota Transfer Therapy on Autism Symptoms and Gut Microbiota

Dae-Wook Kang[1,2,8], James B. Adams[3], Devon M. Coleman[3], Elena L. Pollard[3], Juan Maldonado[1,2], Sharon McDonough-Means[4], J. Gregory Caporaso[5,6], and Rosa Krajmalnik-Brown[1,2,7,*]

[1]Biodesign Swette Center for Environmental Biotechnology, Arizona State University, Tempe, AZ USA 85287

[2]Biodesign Center for Fundamental and Applied Microbiomics, Arizona State University, Tempe, AZ USA 85287

[3]School for Engineering of Matter, Transport and Energy, Arizona State University, Tempe, AZ USA 85287

[4]Integrative Developmental Pediatrics, Tucson, AZ USA 85701

[5]Center for Applied Microbiome Science, Pathogen and Microbiome Institute, Northern Arizona University, Flagstaff, AZ USA 86011

[6]Department of Biological Sciences, Northern Arizona University, Flagstaff, AZ USA 86011

[7]School of Sustainable Engineering and the Built Environment, Arizona State University, Tempe, AZ USA 85287

[8]Current address: Department of Civil and Environmental Engineering, The University of Toledo, Toledo, OH, USA 43606

[*]Correspondence: dr.rosy@asu.edu (R.K.B.)

Email addresses:

daewook.kang@utoledo.edu (Dae-Wook Kang), jim.adams@asu.edu (James B. Adams), devon.coleman@asu.edu (Devon Coleman), elena.pollard@asu.edu (Elena L. Pollard), juan.Maldonadoortiz@asu.edu (Juan Maldonado), mcdosh@dakotacom.net (Sharon McDonough-Means), gregcaporaso@gmail.com (J. Gregory Caporaso), Dr.Rosy@asu.edu (Rosa Krajmalnik-Brown)

Supplementary Figures

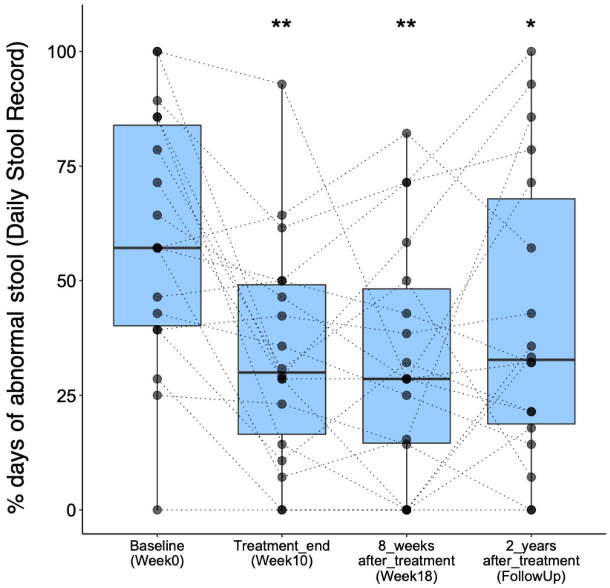

Supplementary Figure S1. Changes in % days of abnormal stool (Daily Stool Record: DSR) of 18 children with ASD at two-year follow-up after treatment stopped. Asterisks (at the top of the box plot) indicate whether individuals (at each time point) have significantly changed since pre-treatment (Week 0 of original Phase 1 trial). Based on two-tailed Wilcoxon signed-rank test, *ns* indicates not significant, single asterisk indicates $p < 0.05$, double asterisks indicate $p < 0.01$, triple asterisks indicate $p < 0.001$.

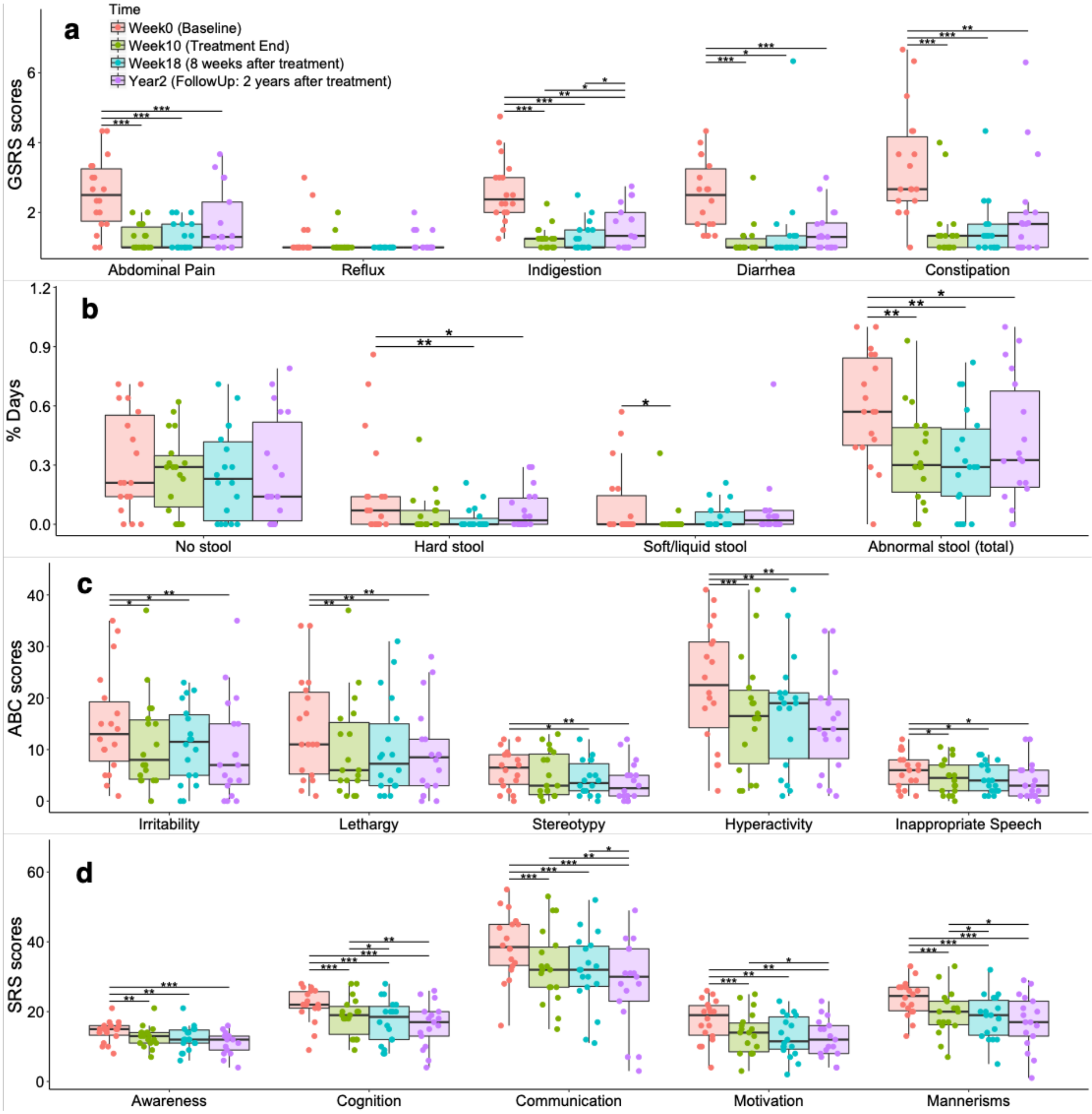

Supplementary Figure S2. **Changes in subscores of GI- and ASD-related symptoms of 18 children with ASD at two-year follow-up after treatment stopped.** Subscales of (**a**) GSRS, (**b**) DSR, (**c**) ABC, and (**d**) SRS at baseline, FMT treatment end, 8 weeks after treatment, and 2 years after treatment. For DSR, based on the Bristol stool form scale, the stool types 1 and 2 were combined as 'hard stool' and types 6 and 7 were as 'soft/liquid stool'. % days of 'abnormal stool' is a sum of % days of 'no stool', 'hard stool', and 'soft/liquid stool'. r*: p<0.05, **: p<0.01, ***: p<0.001 (Wilcoxon signed-rank test).

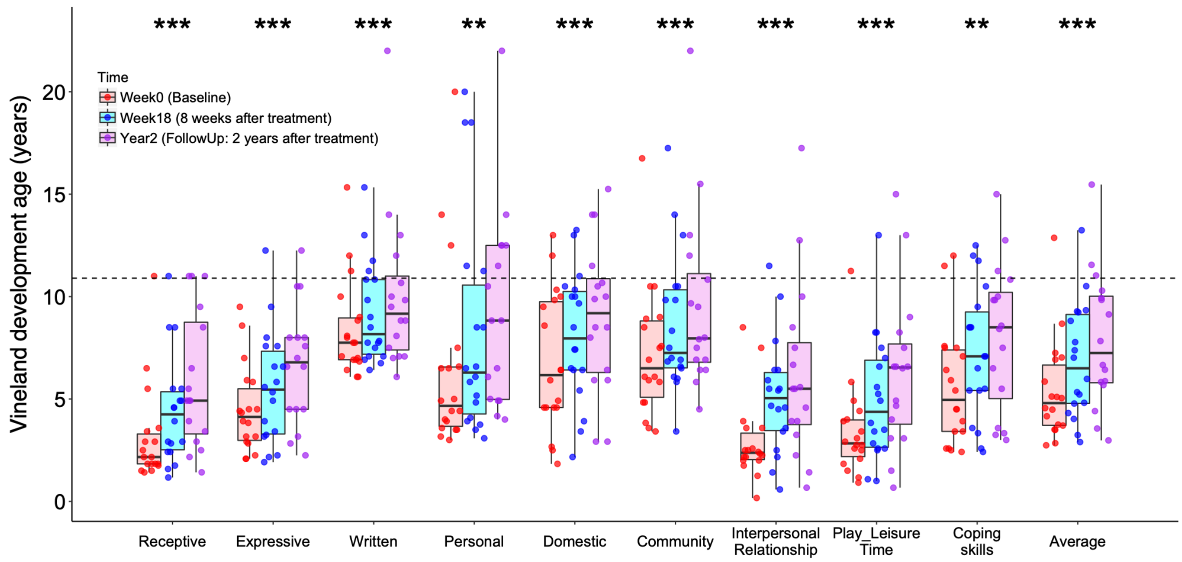

Supplementary Figure S3. Vineland Developmental Age (in years) for individual subscales and for the average of all subscales, measured at baseline, at the end of observation 4 months later, and at two years after the treatment. Note that the average chronological age was 10.9 years at the start of treatment (showed in a dotted line). As seen here, at baseline there were delays in all areas, especially in the core autism areas of language and social (interpersonal) ability. Subscales are under either communication domain (receptive, expressive, and written), or daily living skills domain (personal, domestic, and community), or socialization domain (Interpersonal relationships, play and leisure Time, and coping skills). *: $p<0.05$, **: $p<0.01$, ***: $p<0.001$ (two-tailed Wilcoxon signed-rank test).

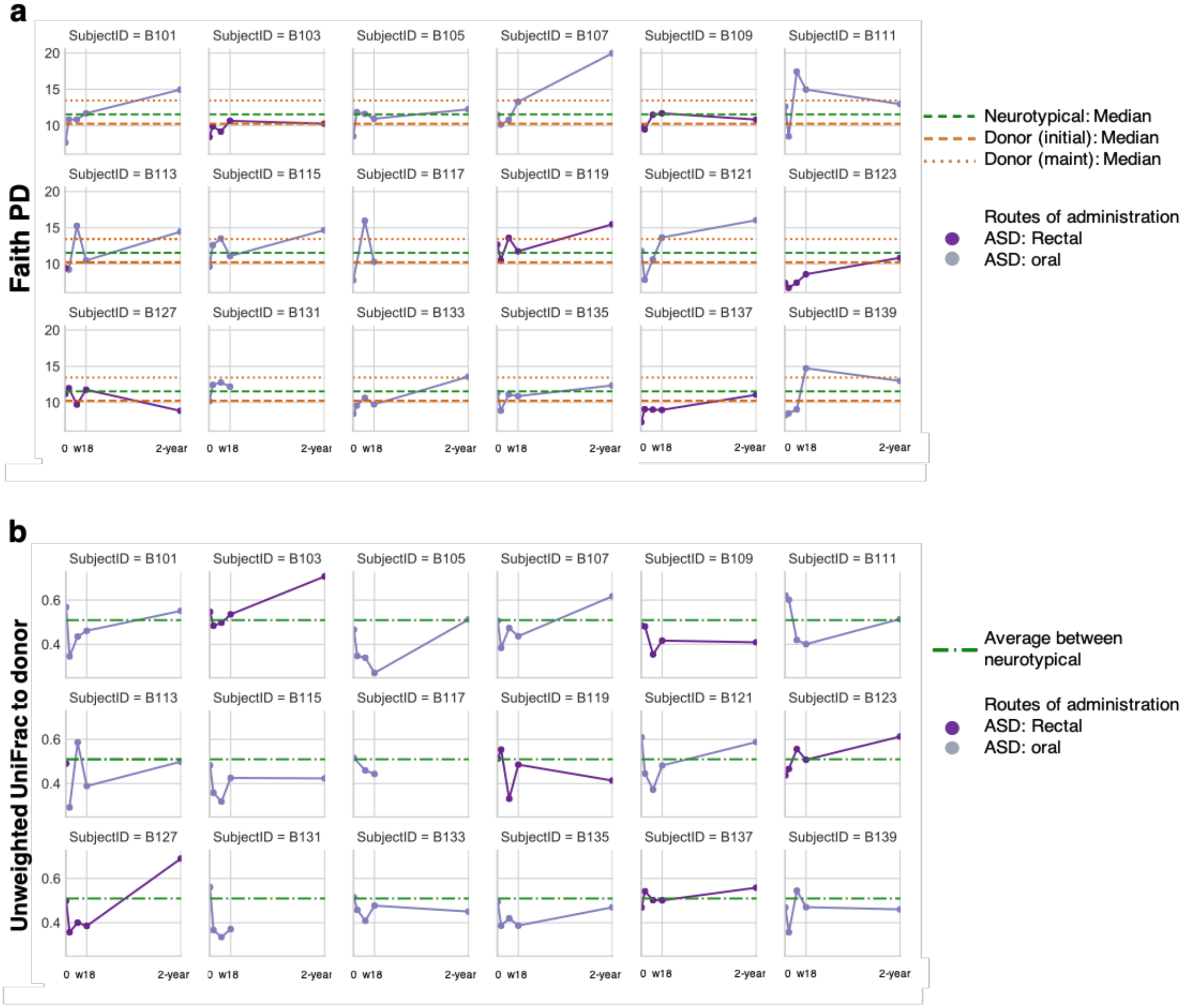

Supplementary Figure S4. Stool microbiota assessments at two-year follow-up after treatment stopped. (**a**) Faith's phylogenetic diversity (PD) tracked on a per individual basis for all MTT recipients. 16 children with ASD were followed up at two-years after treatment stopped. *Orange lines* indicate median PD of the donor samples (*dashed line* represents initial donor samples ($n = 5$), and *dotted line* represents maintenance dose samples ($n = 2$)), and *green line* indicates median PD of 20 neurotypical controls at week 0. (**b**) Unweighted UniFrac distances between ASD gut microbiota and most relevant donor sample on a per individual basis (initial donor sample at weeks 0 and 3, most recent maintenance dose sample at weeks 10 and 18, and 2 years). *Green line* indicates the median interpersonal variation between neurotypical controls and illustrates that prior to treatment the difference in gut microbiota composition between MTT recipients and donors was on the order of normal interpersonal variation. See also Supplementary Fig. S5 and S6.

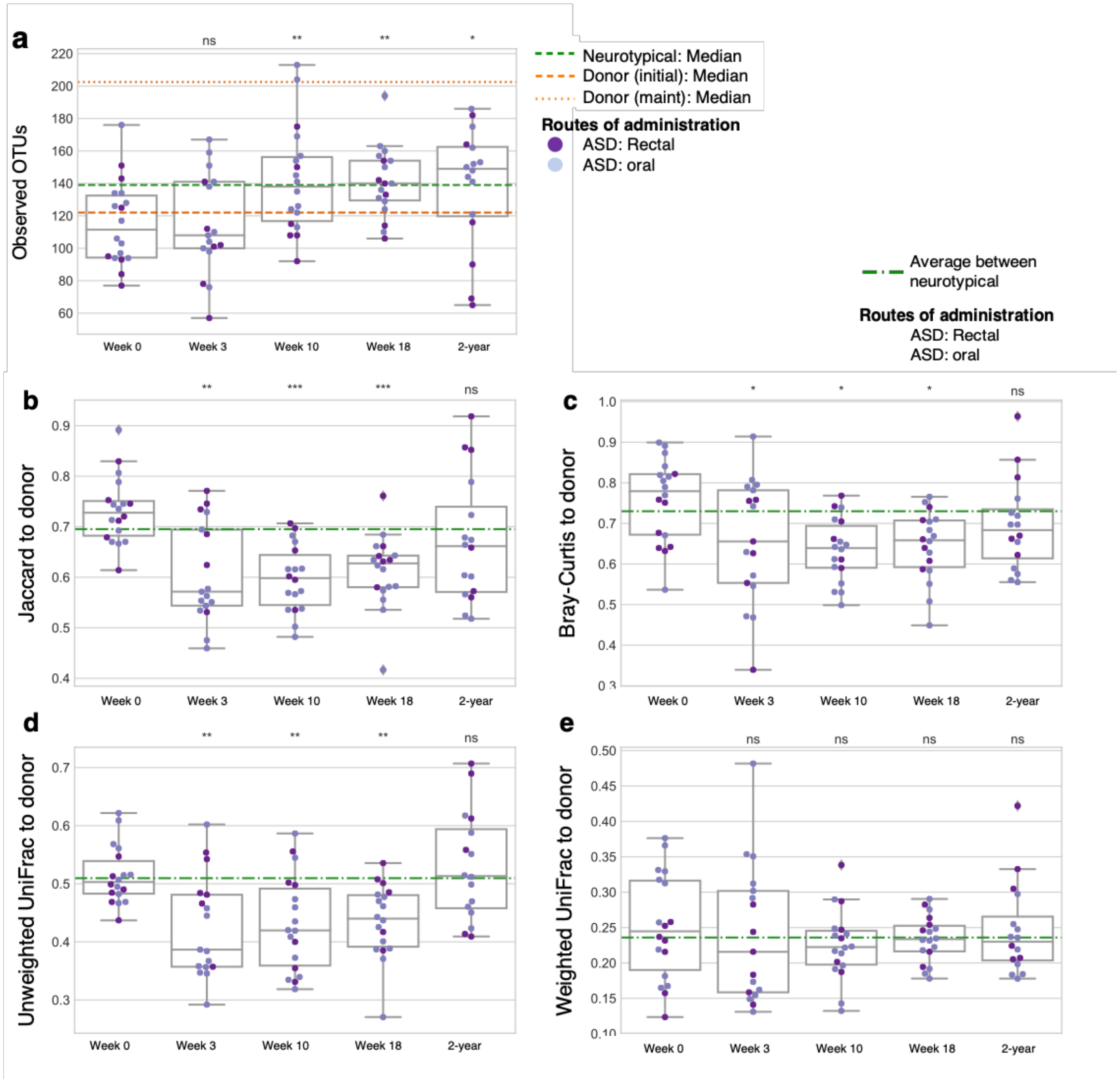

Supplementary Figure S5. Stool microbiota assessments and engraftment analysis at two-year follow-up after treatment stopped. (**a**) Changes in microbial richness, as measured by *Observed OTUs*, a non-phylogenetic diversity metric in the microbiota of 18 children with ASD as measured from stool samples. *Orange lines* indicate median PD of the donor samples (*dashed line* represents initial donor samples ($n = 5$), and *dotted line* represents maintenance dose samples ($n = 2$)), and *green line* indicates median PD of 20 neurotypical controls at week 0. (**b-e**) Engraftment analysis with four diversity metrics of Jaccard, Bray-Curtis, Unweighted UniFrac, and Weighted UniFrac distances between ASD gut microbiota and most relevant donor sample (initial donor sample at weeks 0 and 3, most recent maintenance dose sample at weeks 10 and 18, and 2 years). *Green line* indicates the median interpersonal variation between neurotypical controls and illustrates that prior to treatment the difference in gut microbiota composition between MTT recipients and donors was on the order of normal interpersonal variation. *ns* indicates not significant, *single asterisk* indicates $p < 0.05$, *double asterisks* indicate $p < 0.01$, *triple asterisks* indicate $p < 0.001$ (two-tailed Wilcoxon signed-rank test comparing weeks 3, 10, and 18 and two-year to week 0 values).

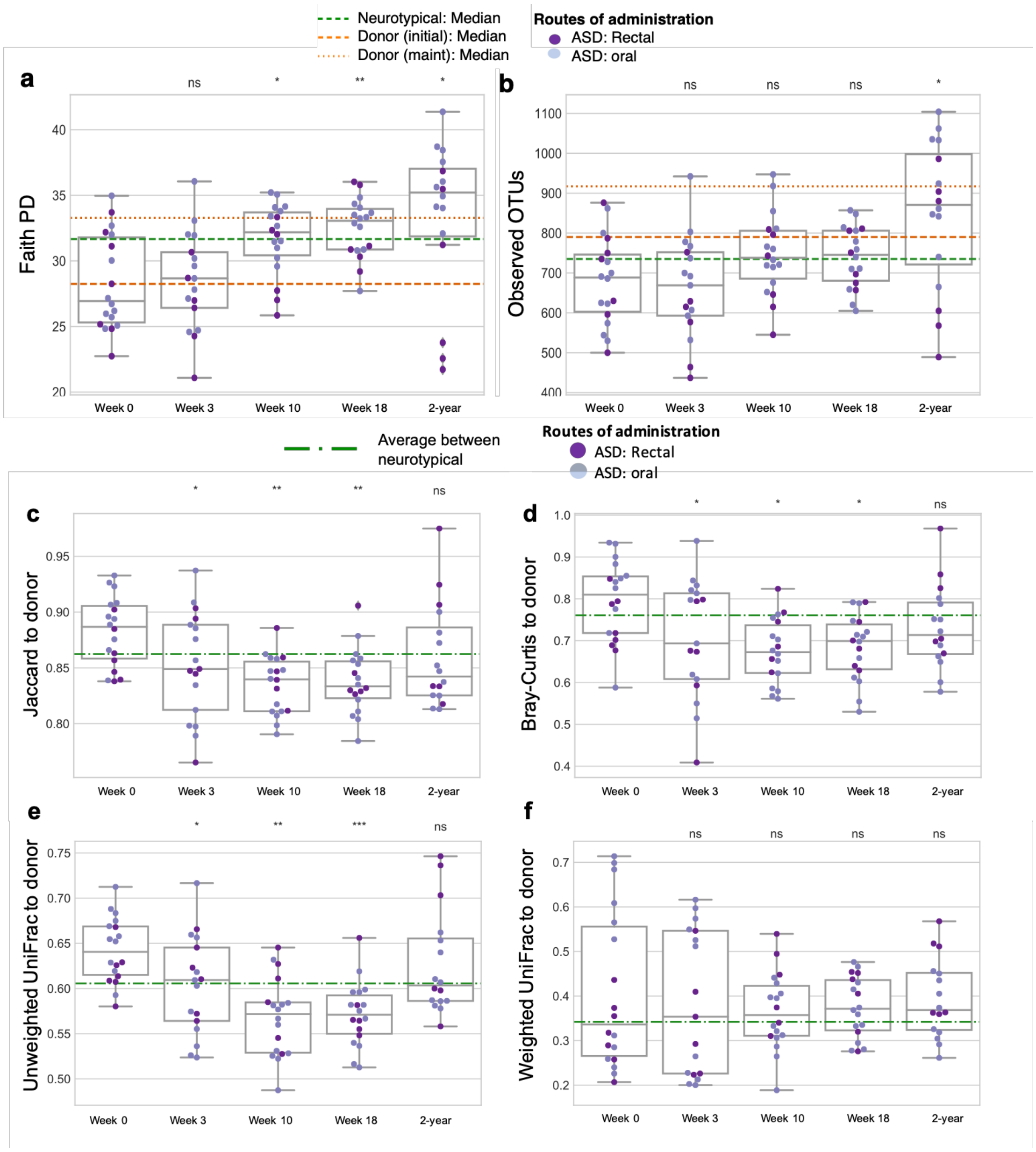

Supplementary Figure S6. Stool microbiota diversity assessments and engraftment analysis using the quality filtering approach described in Bokulich et al. (2013). (**a-b**) *Orange lines* indicate median PD or observed OTUs of the donor samples (*dashed line* represents initial donor samples ($n = 5$), and *dotted line* represents maintenance dose samples ($n = 2$)), and *green line* indicates median PD of 20 neurotypical controls at week 0. (**c-f**) Engraftment analysis with four diversity metrics of Jaccard, Bray-Curtis, Unweighted UniFrac, and Weighted UniFrac distances using the quality filtering approach described in Bokulich et al. (2013). *Green line* indicates the median interpersonal variation between neurotypical controls and illustrates that prior to treatment the difference in gut microbiota composition between MTT recipients and donors was on the order of normal interpersonal variation. *ns* indicates not significant, *single asterisk* indicates $p < 0.05$, *double asterisks* indicate $p < 0.01$, *triple asterisks* indicate $p < 0.001$ (two-tailed Wilcoxon signed-rank test comparing weeks 3, 10, and 18 and two-year to week 0 values).

Supplementary Tables

Supplementary Table S1. A summary of changes to the medications, nutritional supplements, and diets between the end of the original MTT trial and the two-year follow-up since the treatment stopped.

Supplementary Table S2. A dataset of participants' characteristics, GI and behavior assessments, and their medical and diet history.

Supplementary File

Supplementary File S1. The provenance data available with complete details on the bioinformatics methods, including the versions of all software and dependencies, and all commands and parameter settings. This information can be viewed visually at https://view.qiime2.org.
